# Sterility, safety, and preventive efficacy of three polyvalent hyperimmune sera against bacterial infection in a white mouse challenge model

**DOI:** 10.3389/fvets.2026.1810086

**Published:** 2026-04-02

**Authors:** Ibragim Tutkyshbay, Maxat Berdikulov, Zura Yessimsiitova, Abzal Makhmutov, Damir Khussainov, Gulzhan Mussayeva, Raikhan Nissanova, Kuandyk Shynybayev, Flyura Bakiyeva, Dmitriy Taranov

**Affiliations:** 1 Mukhtar Auezov South Kazakhstan University, Shymkent, Kazakhstan; 2 National Veterinary Reference Center, Almaty, Kazakhstan; 3 Al-Farabi Kazakh National University, Almaty, Kazakhstan; 4 Kazakh National Agrarian Research University, Almaty, Kazakhstan; 5 Kazakh Scientific Research Veterinary Institute, LLP, Almaty, Kazakhstan; 6 LLP NPTTS “Zhalyn”, Almaty, Kazakhstan

**Keywords:** *Clostridium perfringens*, *Escherichia coli*, experimental infection, hyperimmune serum, passive immunization, *Pasteurella*, prophylaxis, *Salmonella*

## Abstract

**Introduction:**

Polyvalent hyperimmune sera are widely used for emergency passive immunoprophylaxis; however, controlled experimental evidence supporting their safety and preventive efficacy remains limited.

**Methods:**

In this study, three polyantigenic hyperimmune sera were evaluated using a standardized white mouse bacterial challenge model. Sera were generated through stepwise immunization of donor animals with monovalent and polyvalent antigenic formulations targeting enteric and respiratory pathogens. Sterility and safety were assessed prior to *in vivo* application. Preventive efficacy was evaluated following subcutaneous administration of sera 24 h before experimental infection with enteric bacterial pathogens.

**Results:**

Serological characterization confirmed robust virus- and bacteria-specific humoral responses in donor animals, with end-point antibody titers reaching up to 1:2560 in serum preparations containing viral antigens. All tested sera were sterile and well tolerated, with no adverse clinical effects observed during the monitoring period. Prophylactic administration led to a marked increase in survival compared with untreated controls across multiple bacterial challenge models, with absolute risk reduction values ranging from 40 to 100%. In contrast, native serum collected prior to immunization provided only partial or no protection.

**Discussion:**

Overall, these findings provide controlled experimental evidence demonstrating the safety, immunogenicity, and reproducible preventive efficacy of polyvalent hyperimmune sera in a murine bacterial infection model, supporting their consideration as candidate immunobiological agents for passive protection against bacterial infections under controlled experimental conditions.

## 1 Introduction

Hyperimmune sera represent a class of immunobiological preparations obtained from donor animals after targeted immunization and are used to provide immediate passive protection against infectious agents (1–3). In veterinary medicine, such preparations are primarily used in situations where rapid, short-term immunity is required, including periods of high infection pressure, increased susceptibility in young animals, or when active immunization is not feasible or has not yet elicited a protective immune response (4–7). Polyvalent formulations are therefore of particular interest for supportive and prophylactic use in situations that require rapid passive immunity (8–10).

Despite their long-standing application, experimental evaluation of hyperimmune sera remains essential (11, 12). Variability in antigen composition, antibody titers, and production protocols may influence both safety and preventive performance (13, 14). Consequently, each preparation requires validation under controlled conditions to confirm its sterility, clinical tolerability, and biological activity (15–17). Laboratory animal models provide a standardized framework for such assessments, allowing for a direct comparison of a hyperimmune serum with appropriate controls, while minimizing confounding factors inherent to field studies (18–21).

Assessment of preventive efficacy is most informative when based on clearly defined and biologically unambiguous endpoints. Survival following experimental infection represents a robust outcome measure, particularly in challenge models using titrated lethal doses of pathogens (22–24). When combined with a prophylactic administration regimen, this approach allows for the quantification of the protective potential of serum preparations under standardized conditions. The inclusion of native serum collected prior to hyperimmunization further enables differentiation between baseline antibody-mediated effects and protection attributable to targeted hyperimmune responses (6, 25, 26).

Statistical analysis of survival data in experimental challenge studies requires methods appropriate for small group sizes and binary outcomes (27, 28). Exact tests, such as Fisher’s exact test, are well suited for this purpose, particularly in the presence of extreme outcome distributions, while complementary approaches, including chi-square testing with continuity correction and effect size measures such as absolute risk reduction, support transparent interpretation of preventive benefit (29, 30).

Against this background, the present study aimed to evaluate the sterility, safety, and preventive efficacy of three polyvalent hyperimmune sera using a white mouse bacterial challenge model. By integrating safety assessment, sterility testing, and a standardized prophylactic infection design with survival as the primary endpoint, this study provides controlled experimental evidence to support the rational interpretation of the preventive potential of these serum preparations.

## 2 Materials and methods

### 2.1 Study setting and ethical approval

The study was conducted under controlled laboratory conditions using a white mouse experimental model. All procedures involving animals were performed in accordance with national and institutional guidelines for laboratory animal welfare and were reviewed and approved by the Institutional Bioethics Committee at the Institute of Genetics (Protocol No. 1, approved on 20 October 2025).

### 2.2 Animals

White outbred adult male mice, weighing 20–22 g, were used in the experiments. Animals were housed under standard vivarium conditions with controlled temperature (22 ± 2 °C), a 12-h light/dark cycle, and free access to feed and water. Prior to the start of the study, mice were allowed to acclimatize for at least 7 days.

### 2.3 Hyperimmune sera

Three polyvalent hyperimmune sera were produced by stepwise active immunization of donor cattle under controlled laboratory conditions. Donor animals were clinically healthy and maintained under standard husbandry conditions. The hyperimmunization protocol consisted of repeated administrations of antigen preparations at defined intervals designed to induce a strong secondary humoral immune response. The antigenic formulations comprised epizootically relevant viral and bacterial pathogens associated with enteric and respiratory diseases in young ruminants. Serum No. 1 contained antigens of bovine viral diarrhea virus, bovine herpesvirus 1, parainfluenza virus type 3, rotavirus, *Escherichia coli*, and *Salmonella* spp. Serum No. 2 included antigens of bovine herpesvirus 1, bovine viral diarrhea virus, parainfluenza virus type 3, *Salmonella* spp., and *Pasteurella multocida*. Serum No. 3 comprised antigens associated with clostridial enterotoxemia, including *Clostridium perfringens* types A, C, and D, along with enteric bacterial pathogens.

Hyperimmunization was performed by repeated subcutaneous administration of antigen preparations with gradually increasing doses at defined intervals sufficient to induce a pronounced secondary humoral immune response. Donor animals were clinically monitored throughout the immunization period. Blood samples were collected at predetermined time points, and sera were obtained by standard clotting and centrifugation procedures.

Virus-specific antibody responses to bovine herpesvirus 1, bovine viral diarrhea virus, parainfluenza virus type 3, and rotavirus were assessed in donor animals by enzyme-linked immunosorbent assay (ELISA), while antibodies against *Salmonella* spp. and *Pasteurella multocida* were evaluated using agglutination assays. Only sera demonstrating stable, high titers of specific antibodies were used in subsequent prophylactic efficacy experiments in mice. Native serum collected from donor animals prior to immunization served as a comparator. Antigen preparations were produced using standard inactivation and formulation procedures commonly applied in veterinary immunobiological production.

### 2.4 Sterility testing

Sterility of the serum preparations was assessed using both cell culture-based and bacteriological methods. Samples were inoculated into cell cultures and onto standard bacteriological media and incubated under routine sterility-testing conditions, with daily monitoring performed throughout the observation period. Cell cultures were examined for cytopathic effects, while bacteriological media were assessed for microbial growth. Appropriate uninoculated culture media and cell culture controls were included in parallel.

### 2.5 Safety assessment

Safety was evaluated in mice following a single subcutaneous administration of 0.5 mL of hyperimmune serum in the interscapular region. Animals were observed daily for 10 days for general condition and for clinical signs of intoxication or adverse reactions. Each serum preparation was tested in a separate group of 10 mice under the same observation conditions.

### 2.6 Preventive efficacy challenge design

Preventive efficacy was assessed using an experimental infection model. Mice received a single subcutaneous dose of 0.5 mL of hyperimmune serum 24 h prior to the challenge. Control groups received either native serum or no serum. Experimental infection was performed by intraperitoneal inoculation with titrated lethal doses of *Salmonella dublin*, *Escherichia coli* (K99 and A20), *Pasteurella multocida*, *Clostridium perfringens* types A, C, and D, and *Salmonella* Abortusovis, as indicated in Tables 1–3. The challenge doses corresponded to titrated lethal challenge doses established in preliminary experiments to ensure consistent mortality in untreated control animals. Final survival was recorded at the end of the post-challenge observation period. Animals were monitored daily for clinical condition and survival until the experimental endpoint.

**Table 1:** Preventive efficacy of serum no. 1 in white outbred mice after experimental infection with enteric bacterial pathogens (*n* = 10 per group).

Pathogen	Comparison groups	ARR,* %	*p* (Fisher’s exact test)	*p* (*χ*^2^ test with Yates’ correction)
*Salmonella dublin*	Hyperimmune vs. Native serum	40	0.524	0.519
*Salmonella dublin*	Hyperimmune vs. No-serum	80	0.0476	0.0528
*Escherichia coli* K99	Hyperimmune vs. Native serum	60	0.206	0.206
*Escherichia coli* K99	Hyperimmune vs. No-serum	80	0.0476	0.0528
*Escherichia coli* A20	Hyperimmune vs. Native serum	80	0.0476	0.0528
*Escherichia coli* A20	Hyperimmune vs. No-serum	100	0.008	0.011

Absolute risk reduction (ARR) denotes the absolute reduction in mortality calculated as the difference in mortality rates between the comparator group and the hyperimmune serum group. Survival is expressed as the percentage of surviving animals in each group. Statistical significance was assessed using Fisher’s exact test; *χ*^2^ test with Yates’ correction is provided for reference.

**Table 2:** Preventive efficacy of serum no. 2 in white outbred mice after experimental infection with *Salmonella dublin* and *Pasteurella multocida* (*n* = 10 per group).

Pathogen	Comparison groups	ARR, %	*p* (Fisher’s exact test)	*p* (*χ*^2^ test with Yates’ correction)
*Salmonella dublin*	Hyperimmune vs. Native serum	100	0.00794	0.0114
*Salmonella dublin*	Hyperimmune vs. No-serum	100	0.00794	0.0114
*Pasteurella multocida*	Hyperimmune vs. Native serum	80	0.0476	0.0528
*Pasteurella multocida*	Hyperimmune vs. No-serum	100	0.00794	0.0114

**Table 3:** Preventive efficacy of serum no. 3 against bacterial pathogens in white outbred mice (*n* = 10 animals per group).

Pathogen	Comparison groups	ARR, %	*p* (Fisher’s exact test)	*p* (*χ*^2^ test with Yates’ correction)
*Clostridium perfringens* type A	Hyperimmune vs. Native serum	80	0.048	0.053
*Clostridium perfringens* type A	Hyperimmune vs. No-serum	100	0.008	0.011
*Clostridium perfringens* type C	Hyperimmune vs. Native serum	80	0.048	0.053
*Clostridium perfringens* type C	Hyperimmune vs. No-serum	80	0.048	0.053
*Clostridium perfringens* type D	Hyperimmune vs. Native serum	80	0.048	0.053
*Clostridium perfringens* type D	Hyperimmune vs. No-serum	80	0.048	0.053
*Escherichia coli* K99	Hyperimmune vs. Native serum	60	0.167	0.168
*Escherichia coli* K99	Hyperimmune vs. No-serum	100	0.008	0.011
*Escherichia coli* A20	Hyperimmune vs. Native serum	60	0.206	0.206
*Escherichia coli* A20	Hyperimmune vs. No-serum	80	0.048	0.053
*Salmonella* Abortusovis	Hyperimmune vs. Native serum	60	0.206	0.206
*Salmonella* Abortusovis	Hyperimmune vs. No-serum	80	0.048	0.053

ARR, calculated as the difference in mortality rates between the comparator group and the hyperimmune serum group. Survival is expressed as the percentage of surviving animals in each group. Statistical significance was assessed using Fisher’s exact test; *χ*^2^ test with Yates’ correction is provided for reference.

### 2.7 Statistical analysis

Survival data were analyzed using Fisher’s exact test as the primary statistical method, given the small group sizes and extreme outcome proportions. Pearson’s chi-square test with Yates’ continuity correction was applied as a confirmatory approach. Absolute risk reduction (ARR) was calculated to estimate the magnitude of the preventive effect. Differences were considered statistically significant at a *p*-value of < 0.05. Survival outcomes are presented as percentages, with group sizes indicated in the corresponding table titles (*n* = 10 animals per group). Given the exploratory preclinical design of the study, the statistical analysis was intended to support a comparative interpretation of prophylactic effects across challenge models rather than formal adjustment for multiplicity across all pathogen-specific comparisons.

## 3 Results

### 3.1 Serological characterization of Serum No. 2

Serological analysis confirmed the successful induction of pathogen-specific humoral immune responses in donor animals following stepwise immunization. ELISA demonstrated high titers of virus-specific antibodies against bovine herpesvirus 1, bovine viral diarrhea virus, parainfluenza virus type 3, and rotavirus. Endpoint antibody titers reached 1:2560, indicating a pronounced antiviral antibody response.

**Figure 1:**
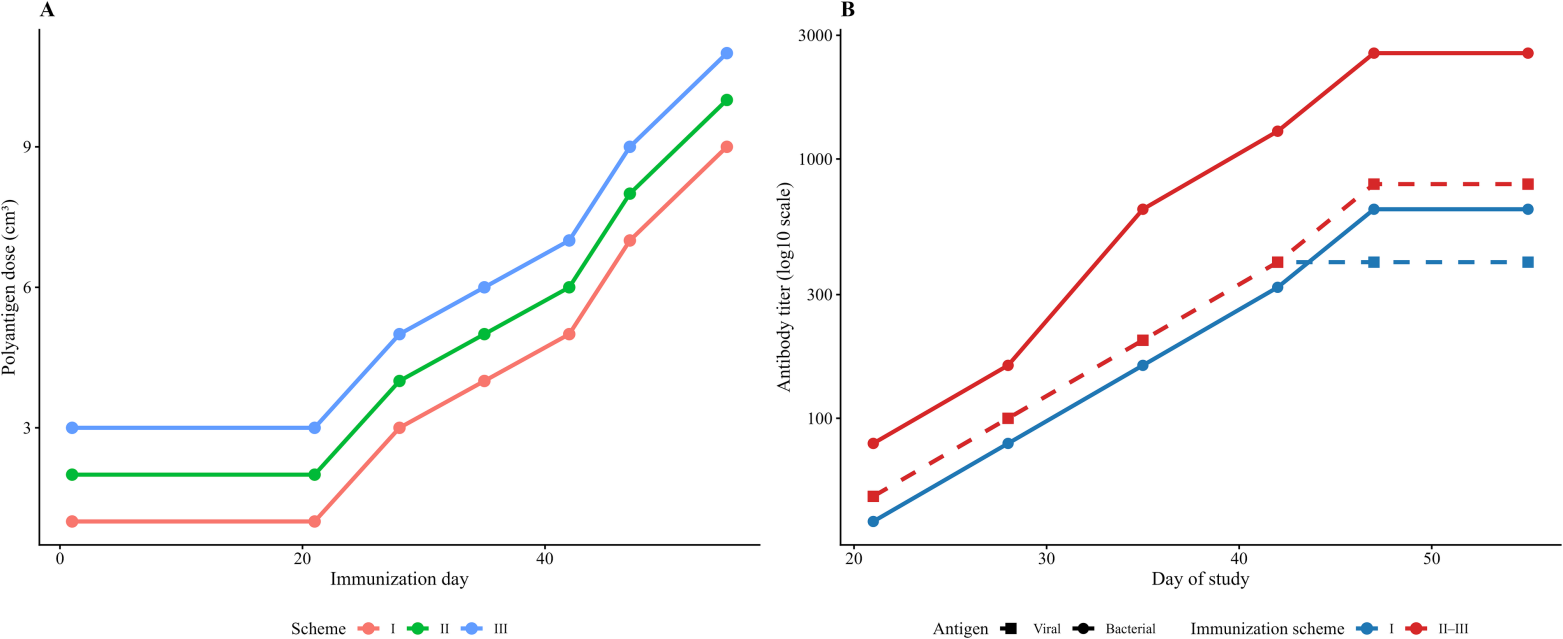
Dynamics of polyantigen administration and specific antibody titers in donor cattle during stepwise immunization. **(A)** Stepwise polyantigen dosing schedule by immunization day for schemes I–III; the dosing regimen was identical for the bacterial and viral antigen complexes and is therefore shown once. **(B)** Changes in specific antibody titers over time against bacterial (solid lines) and viral (dashed lines) antigens measured during the study period for scheme I and schemes II–III (identical dynamics).

Antibody activity against bacterial antigens, including *Salmonella* spp. and *Pasteurella multocida*, was confirmed using standard agglutination assays, with positive titers detected across all tested serum preparations. In contrast, native sera collected prior to immunization did not demonstrate specific reactivity to either viral or bacterial antigens and remained at background levels. A summary of the serological findings is presented in Table 4. Figure 1 provides an overview of the stepwise polyantigen dosing schedule used for hyperimmunization (identical for bacterial and viral antigen complexes) and the corresponding dynamics of specific antibody titers in donor cattle over the immunization period. The temporal pattern illustrates a progressive increase in antibody levels following dose escalation and booster administrations, consistent with the development of a pronounced secondary humoral response.

**Table 4:** Serological characterization of polyvalent hyperimmune sera obtained by stepwise immunization (ELISA and agglutination assays).

Serum No. (donor cattle)	Target antigen(s)	Assay method	Time point (day)	Antibody titer
Serum 2	BoHV-1, BVDV, PI-3, Rotavirus	ELISA	21	1:40–1:80
Serum 2	*Salmonella* spp., *Pasteurella multocida*	Agglutination	21	1:50
Serum 2	BoHV-1, BVDV, PI-3, Rotavirus	ELISA	35	1:160–1:640
Serum 2	*Salmonella* spp., *Pasteurella multocida*	Agglutination	35	1:200
Serum 2	BoHV-1, BVDV, PI-3, Rotavirus	ELISA	47–55	up to 1:2560
Serum 2	*Salmonella* spp., *Pasteurella multocida*	Agglutination	47–55	up to 1:800
Native serum	All antigens	ELISA/Agglutination	—	Negative

Antibody titers are presented as endpoint dilutions, confirming the presence of both virus- and bacteria-specific antibodies in Serum No. 2. These results demonstrate the effectiveness of the applied immunization protocol and provide a clear immunological basis for the subsequent *in vivo* evaluation of prophylactic efficacy.

Serological testing was not performed for Serum No. 1 and Serum No. 3 because their antigenic components were predominantly bacterial.

### 3.2 Sterility and safety

Sterility testing confirmed that all tested hyperimmune serum preparations were free of microbial contamination. No bacterial growth was detected on bacteriological media after incubation, and no cytopathic changes were observed in cell culture (Vero/MDCK) during the observation period. In the safety experiment, all mice remained clinically normal throughout the 10-day monitoring period following subcutaneous administration of 0.5 mL of the respective hyperimmune serum. No signs of disease, intoxication, or adverse reactions were recorded.

### 3.3 Preventive efficacy of Serum No. 1 (*Escherichia coli*/*Salmonella* model)

Prophylactic administration of Serum No. 1 24 h before the experimental infection increased survival compared with the no-serum control (Table 1). Following a challenge with *Salmonella dublin* and *Escherichia coli* K99, survival in the hyperimmune serum group reached 80%, whereas no animals survived in the no-serum control groups. For *E. coli* A20, survival was 100% in the hyperimmune serum group and 0% in the no-serum control group. Compared with native serum, survival was higher in the hyperimmune serum group across pathogens; however, statistical significance for hyperimmune vs. native serum comparisons was pathogen-dependent. In contrast, comparisons of hyperimmune serum vs. the no-serum control were statistically significant for all pathogens tested (Fisher’s exact test, *p* < 0.05). Overall, these results indicate a preventive effect of Serum No. 1 against enteric bacterial pathogens, particularly in comparison with the no-serum control.

### 3.4 Preventive efficacy of serum no. 2 (*Salmonella*/*Pasteurella* model)

Prophylactic administration of Serum No. 2 resulted in increased survival following experimental infection with *Salmonella dublin* and *Pasteurella multocida* (Table 2). For *Salmonella dublin*, survival reached 100% in the hyperimmune serum group, whereas no animals survived in either the native serum or the no-serum control groups. For *Pasteurella multocida*, survival was 100% in the hyperimmune serum group compared with 20% in the native serum group and 0% in the no-serum controls. These outcomes corresponded to high ARR values, reflecting a substantial reduction in mortality following prophylactic serum administration. Statistically significant differences were observed in comparisons between the hyperimmune serum and no-serum control groups for both pathogens (Fisher’s exact test, *p* < 0.05). Comparisons with native serum were statistically significant for *Salmonella dublin* and demonstrated a trend toward higher survival for *Pasteurella multocida*. Overall, these results indicate a preventive effect of Serum No. 2 against enteric and respiratory bacterial infections under the experimental conditions used.

### 3.5 Preventive efficacy of serum no. 3 (enteric/Clostridial model)

Prophylactic administration of Serum No. 3 prior to experimental infection resulted in high survival rates across all tested bacterial challenge models, including *Clostridium perfringens* types A, C, and D, along with enteric pathogens (*Escherichia coli* K99, *E. coli* A20, and *Salmonella* Abortusovis) (Table 3).

In comparison with no-serum control groups, survival in serum-treated mice ranged from 80 to 100%, whereas no survival (0%) was observed in all corresponding control groups. This translated into an ARR of 80–100%, with statistically significant differences consistently detected (Fisher’s exact test, *p* < 0.05). Comparisons between hyperimmune serum and native serum controls demonstrated pathogen-dependent effects. For *Clostridium perfringens* types A, C, and D, survival rates in the hyperimmune serum groups ranged from 80 to 100%, compared with 0–20% in native serum controls, corresponding to ARR values of 80%. These differences reached or approached statistical significance depending on the pathogen. For enteric bacteria, prophylactic efficacy against *E. coli* K99 and *E. coli* A20 was evident, with ARR values of 60–100%. While comparisons with no-serum controls were statistically significant, differences relative to native serum controls were not uniformly significant. A similar pattern was observed for *Salmonella* Abortusovis, where hyperimmune serum administration resulted in 80% survival compared with 0–20% in control groups. Collectively, these findings indicate a robust and reproducible preventive effect of Serum No. 3 across multiple bacterial challenge models, with the strongest and most consistent protection observed relative to no-serum controls under the experimental conditions used.

### 3.6 Integrated visualization of survival outcomes

**Figure 2:**
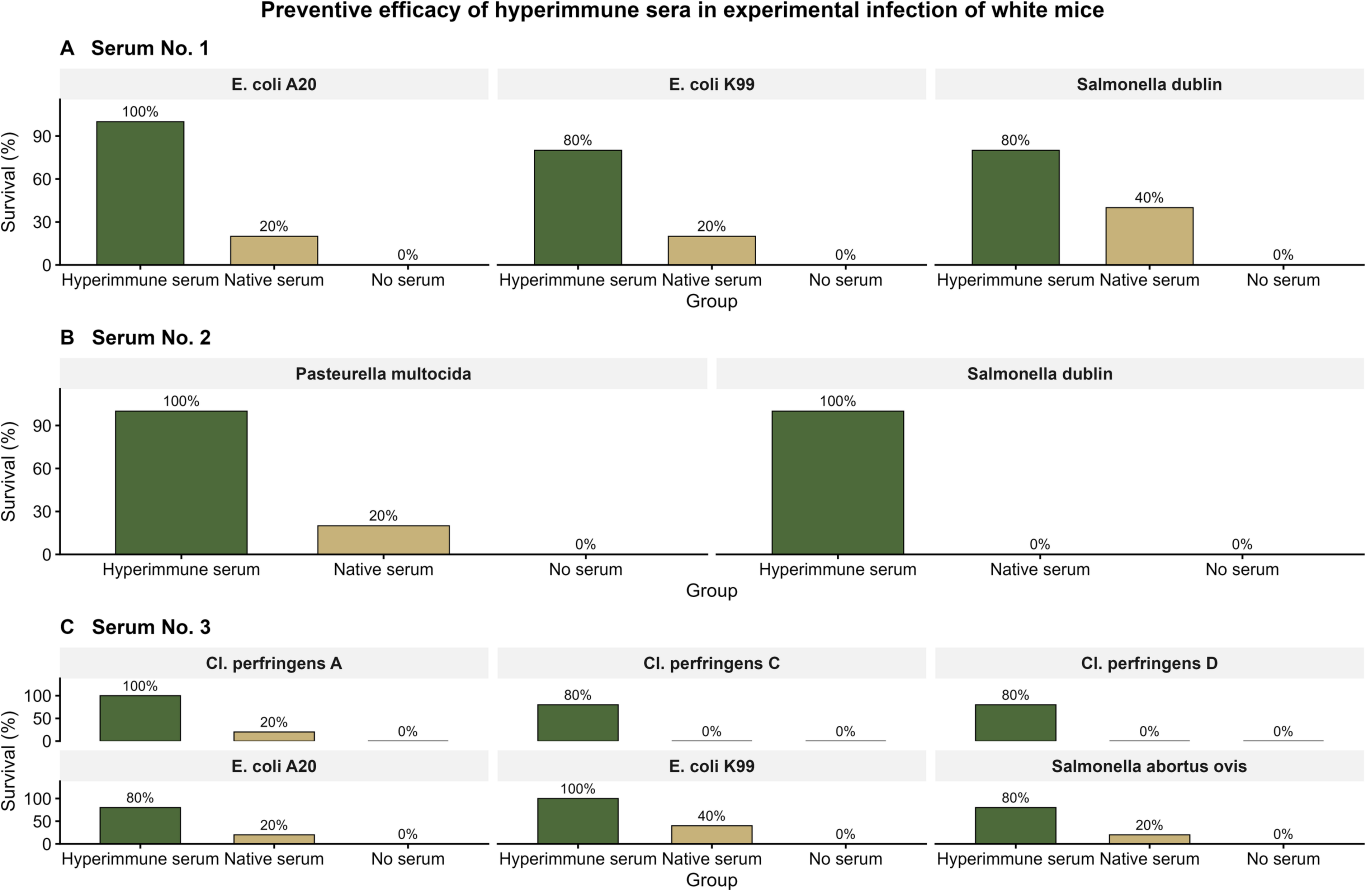
Survival (%) of white mice after experimental infection following prophylactic administration of hyperimmune serum compared with native serum and no-serum controls. **(A)** Enteric bacterial challenge models (*Escherichia coli* A20, *Escherichia coli* K99, *Salmonella dublin*); **(B)** *Salmonella dublin* and *Pasteurella multocida* challenge models; **(C)** clostridial and enteric bacterial challenge models, including *Clostridium perfringens* types **A**, **C**, and **D**, *Escherichia coli* A20, *Escherichia coli* K99, and *Salmonella Abortusovis*.

A consolidated graphical representation of survival outcomes is presented in Figure 2. Across all experimental challenge models included in this study, prophylactic administration of hyperimmune serum was consistently associated with markedly higher survival relative to no-serum controls, whereas comparisons with native serum showed variable, pathogen-dependent differences. Native serum conferred only limited protection, whereas the absence of serum was uniformly associated with minimal or absent survival following experimental infection.

## 4 Discussion

This study provides controlled experimental evidence that three polyvalent hyperimmune sera are sterile, well-tolerated in a murine model and are capable of conferring reproducible prophylactic protection against bacterial challenge when administered prior to infection. Importantly, these conclusions are supported by an integrated experimental design combining sterility assessment, safety evaluation, serological characterization, and preventive efficacy testing, with survival used as a clearly defined and clinically relevant endpoint (31).

The safety profile of the tested sera was consistent across all experimental series. No clinical signs of intoxication, adverse reactions, or behavioral abnormalities were observed during the 10-day monitoring period following subcutaneous administration. These findings indicate that the applied dose and route of administration were well-tolerated under laboratory conditions. In parallel, sterility testing confirmed the absence of microbial contamination and cytopathic effects, supporting the suitability of the preparations for *in vivo* experimental use.

Serological analysis provided direct immunological validation of the hyperimmunization protocol. High titers of virus-specific antibodies were detected in Serum No. 2 against bovine herpesvirus 1, bovine viral diarrhea virus, parainfluenza virus type 3, and rotavirus, with endpoint titers reaching up to 1:2560, indicating a robust and stable humoral immune response. In addition, antibody activity against bacterial antigens, including *Salmonella* spp. and *Pasteurella multocida*, was confirmed by agglutination assays. In contrast, native sera collected prior to immunization showed no specific reactivity, supporting the antigen-specific nature of the induced immune response. Serological testing was not performed on Serum No. 1 and Serum No. 3 due to the predominantly bacterial composition of their antigenic formulations, for which agglutination-based assessment is methodologically appropriate.

No indications of antigenic interference were observed during the stepwise immunization process, as antibody titers against both viral and bacterial antigen complexes increased progressively in donor cattle. However, the present study did not specifically investigate phenomena such as original antigenic sin or antigenic competition, and dedicated immunological studies would be required to evaluate these mechanisms in detail.

Preventive efficacy was evaluated using a prophylactic regimen in which hyperimmune sera were administered 24 h prior to experimental infection with defined bacterial pathogens. The 24-h interval was selected to allow sufficient time for systemic distribution of passively transferred antibodies prior to pathogen exposure, a design commonly used in experimental passive immunization models. Across all challenging models, hyperimmune serum administration resulted in a marked increase in survival compared with no-serum controls, with absolute risk reduction values ranging from 40 to 100%. Statistically significant differences were consistently observed in comparisons with untreated controls, while comparisons with native serum demonstrated pathogen-dependent effects. Native serum provided only partial protection, typically associated with low baseline levels of non-specific antibodies, highlighting the added value of targeted hyperimmunization.

Although high antibody titers were detected in hyperimmunized donor cattle, the present study did not aim to establish quantitative correlates of protection against the respective pathogens in targeted livestock species. In this study, mice were used as a controlled preclinical recipient model for passive serum transfer and challenge. Therefore, the measured donor antibody titers should be interpreted as indicators of successful hyperimmunization rather than as direct predictors of protective threshold levels in large animal hosts. Establishing protective antibody levels in target livestock species will require dedicated target-species studies.

The applied statistical approach was tailored to the data structure and the experimental design. Fisher’s exact test was appropriate for the analysis of binary survival outcomes in small experimental groups, while Pearson’s *χ*^2^ test with Yates’ correction served as a conservative confirmatory method. It should also be noted that the experimental groups were small, which is typical for controlled murine challenge studies. While survival was used as a robust, biologically unambiguous endpoint for preventive efficacy, additional outcome measures such as bacterial burden or histopathological changes could provide further insight in future studies. The inclusion of absolute risk reduction as an effect size metric complements *p*-value-based inference and allows for a quantitative assessment of the magnitude of the prophylactic benefit, strengthening the internal consistency of the conclusions. The chi-square test with Yates’ correction was included as a conservative confirmatory approach, whereas Fisher’s exact test was considered the primary method for the interpretation of binary outcomes in small groups.

Several limitations should be acknowledged. First, the experiments were conducted in a mouse model, and extrapolation of the results to target livestock species should be approached with caution. Second, group sizes were limited, as they are typical for controlled laboratory challenge experiments, which may reduce statistical power in comparisons involving native serum. Third, the analysis focused on survival as a terminal endpoint, without time-to-event modeling or assessment of immunological correlates in recipient animals. These limitations do not invalidate the observed effects but define the scope within which the findings should be interpreted.

The present study was conducted in a murine experimental model, which provides a standardized and widely used platform for the controlled preclinical evaluation of immunobiological preparations. While murine systems cannot fully reproduce host–pathogen interactions in target livestock species, they enable a comparative assessment of safety and prophylactic efficacy under highly controlled experimental conditions. Such models are commonly used as an initial step in the biological validation of passive immunization strategies prior to subsequent evaluation in target animal species.

Within this framework, the present study should be viewed as a stepwise preclinical validation, providing a controlled experimental basis for subsequent evaluation of polyvalent hyperimmune sera in target animal species and under field-relevant conditions.

Within these boundaries, the present results support the practical rationale for considering polyvalent hyperimmune sera as a supportive immunobiological intervention, particularly in situations requiring immediate passive protection and rapid reduction of infectious losses (32).

## 5 Conclusion

The present study demonstrates that the three evaluated polyvalent hyperimmune sera are sterile, well-tolerated, and provide reproducible prophylactic protection in a murine bacterial challenge model. Stepwise hyperimmunization induced pathogen-specific humoral immune responses, as confirmed by ELISA and agglutination assays, validating the immunogenicity of the applied antigen formulations.

Prophylactic administration of hyperimmune sera 24 h prior to experimental infection significantly increased survival compared with no-serum controls across multiple bacterial pathogens, with large absolute reductions in mortality. In contrast, native serum samples collected prior to immunization provided only partial protection. Collectively, these findings support the potential of polyvalent hyperimmune sera as a supportive immunobiological approach to rapid passive protection against bacterial infections under controlled experimental conditions.

## Data Availability

The original contributions presented in the study are included in the article/supplementary material, further inquiries can be directed to the corresponding author/s.
